# Downregulation of the histone methyltransferase SETD2 promotes imatinib resistance in chronic myeloid leukaemia cells

**DOI:** 10.1111/cpr.12611

**Published:** 2019-05-03

**Authors:** Yaru Sheng, Zhongzhong Ji, Huifang Zhao, Jinming Wang, Chaping Cheng, Weimin Xu, Xue Wang, Yuman He, Kaiyuan Liu, Li Li, Thibault Voeltzel, Veronique Maguer‐Satta, Wei‐Qiang Gao, Helen He Zhu

**Affiliations:** ^1^ State Key Laboratory of Oncogenes and Related Genes, Renji‐Med‐X Stem Cell Research Center, Department of Urology, Ren Ji Hospital, School of Medicine Shanghai Jiao Tong University Shanghai China; ^2^ School of Biomedical Engineering, Med‐X Research Institute Shanghai Jiao Tong University Shanghai China; ^3^ Department of Colorectal Surgery, Xin‐Hua Hospital, School of Medicine Shanghai Jiao Tong University Shanghai China; ^4^ CRCL, Inserm U1052‐CNRS UMR5286 Centre Léon Bérard Lyon France

**Keywords:** chronic myeloid leukaemia, epigenetic regulator, imatinib resistance, SETD2

## Abstract

**Objectives:**

Epigenetic modifiers were important players in the development of haematological malignancies and sensitivity to therapy. Mutations of SET domain‐containing 2 (SETD2), a methyltransferase that catalyses the trimethylation of histone 3 on lysine 36 (H3K36me3), were found in various myeloid malignancies. However, the detailed mechanisms through which SETD2 confers chronic myeloid leukaemia progression and resistance to therapy targeting on BCR‐ABL remain unclear.

**Materials and methods:**

The level of SETD2 in imatinib‐sensitive and imatinib‐resistant chronic myeloid leukaemia (CML) cells was examined by immunoblotting and quantitative real‐time PCR. We analysed CD34^+^CD38^−^ leukaemic stem cells by flow cytometry and colony formation assays upon SETD2 knockdown or overexpression. The impact of SETD2 expression alterations or small‐molecule inhibitor JIB‐04 targeting H3K36me3 loss on imatinib sensitivity was assessed by IC50, cell apoptosis and proliferation assays. Finally, RNA sequencing and ChIP‐quantitative PCR were performed to verify putative downstream targets.

**Results:**

SETD2 was found to act as a tumour suppressor in CML. The novel oncogenic targets MYCN and ERG were shown to be the direct downstream targets of SETD2, where their overexpression induced by SETD2 knockdown caused imatinib insensitivity and leukaemic stem cell enrichment in CML cell lines. Treatment with JIB‐04, an inhibitor that restores H3K36me3 levels through blockade of its demethylation, successfully improved the cell imatinib sensitivity and enhanced the chemotherapeutic effect.

**Conclusions:**

Our study not only emphasizes the regulatory mechanism of SETD2 in CML, but also provides promising therapeutic strategies for overcoming the imatinib resistance in patients with CML.

## 1 INTRODUCTION

Chronic myeloid leukaemia (CML) is a myeloproliferative disorder caused by the malignant transformation of hematopoietic stem cells (HSCs) through *BCR‐ABL* oncogene initiation.^1^ Resulting from a t(9,22) (q34;q11) chromosome translocation, the oncogene encodes a chimeric oncoprotein with constitutive tyrosine kinase activity.^2^, ^3^, ^4^ Imatinib, a classical tyrosine kinase inhibitor (TKI) that specifically targets the *BCR‐ABL* oncogene, has been a front‐line drug for the clinical treatment of CML, leading to cytogenetic and molecular remission of the disease.^5^, ^6^, ^7^, ^8^, ^9^ However, approximately 90% of treated patients ultimately develop imatinib resistance, resulting in disease relapse and poor outcomes.^10^, ^11^, ^12^ Approximately 50% of the CML cases with imatinib resistance have been proven to be caused by BCR‐ABL kinase domain mutations (including T315I, Q252H, G250E, E255K/V and Y253H) as well as locus amplification,^10^, ^13^, ^14^ which can be relatively well cured by second‐generation (Dasatinib, Nilotinib, and Bosutinib) and third‐generation (Ponatinib) TKIs.^15^, ^16^, ^17^ Additionally, the primary resistance driven by leukaemic stem cells (LSCs) has turned out to be a troublesome challenge, demanding prompt solutions.^18^, ^19^, ^20^, ^21^ With their traits of self‐renewal, quiescence and reduced differentiation,^19^, ^20^ the LSCs derived from the *BCR‐ABL*‐initiated malignant transformation of HSCs show *BCR‐ABL* ‐independent behaviour,^10^, ^22^ a fact that is exemplified by the failure of single TKI treatments to eliminate these cells.^23^ Therefore, the exploration of potential targets of LSCs and the generation of novel therapeutic approaches for their specific eradication would significantly benefit the outcomes of patients with CML.

Epigenetic modifiers are involved in various myeloid malignancies and in normal hematopoiesis. For example, DNA methyltransferase 1 (DNMT1), DNMT3A and DNMT3B play key roles in uniquely regulating the differentiation of hematopoietic stem cells and progenitor cells.^24^, ^25^, ^26^, ^27^, ^28^ Meanwhile, genetic alterations through DNA methylation (DNMT3A, TET2 and IDH1/2) and histone modifications (EZH2, ASXL1, KMT2A, CREBBP and HDAC2/3) are found in all types of myeloid haematological disorders.^29^, ^30^ Histone deacetylations have been recently supposed to exert a pivotal role in leukemogenesis, as exemplified by the emergence of histone deacetylase inhibitors as therapeutic measures for targeting LSCs.^20^, ^31^

SET domain‐containing 2 (SETD2) is the major mammalian methyltransferase responsible for catalysing the trimethylation of histone 3 on lysine 36 (H3K36me3).^32^ Mutations of SETD2 have been found in various types of tumours, such as clear cell renal cell carcinoma,^33^, ^34^ breast cancer,^35^, ^36^ glioma,^37^ acute leukaemia and chronic lymphocytic leukaemia.^38^, ^39^ In the recent decades, research studies on the loss‐of‐function mutations of SETD2 have been carried out to investigate the initiation and propagation of acute leukaemia by equipping LSCs with increased self‐renewal potential.^38^, ^40^ Specifically, the downregulation of SETD2 was shown to contribute to chemotherapeutic resistance in MLL‐AF9 fusion protein‐associated leukaemia.^41^ In mouse models with SETD2 specifically depleted, the loss of the methyltransferase disrupted normal hematopoiesis through the impairment of hematopoietic stem cell differentiation, thereby further facilitating their malignant transformation.^42^, ^43^

Herein, we demonstrate that the downregulation of SETD2 facilitates imatinib resistance in CML cells, with LSC marker upregulation, which could be successfully rescued by SETD2 overexpression. Additionally, by restoring the H3K36me3 level through treatment with JIB‐04 (a small‐molecule inhibitor of H3K36me3 demethylase^41^), the sensitivity of CML cells towards imatinib was effectively increased, providing a potential therapeutic strategy to overcome imatinib‐resistant CML.

## 2 MATERIALS AND METHODS

### 2.1 Cell culture and drug treatment

The TF1‐BA, TF1‐BAR, KCL‐22‐sensitive (KCL‐22‐S) and KCL‐22‐resistant (KCL‐22‐R) human CML cell lines, all kind gifts from Professor Veronique Maguer‐Satta (Lyon Cancer Center, France), were cultured in RPMI‐1640 medium supplemented with 10% foetal bovine serum, 2 nmol/L glutamine, 100 U/mL penicillin, 100 μg/mL streptomycin and 20 ng/mL granulocyte‐macrophage colony‐stimulating factor. These cells were authenticated by Shanghai Biowing Applied Biotechnology Co. Ltd. (http://www.biowing.com.cn) through the short tandem repeat (STR) genetic analysis in February 2019. All other chemical reagents used in this study are listed in Supporting Information Table S1.

### 2.2 Construction of the shRNA and *SETD2*‐overexpressing adenoviral vector

The short hairpin RNA (shRNA) lentiviral vectors targeting *SETD2, ERG* and *MYCN* were respectively constructed to establish stable knockdown cell lines in TF1‐BA and KCL‐22‐S cells, with an empty vector as a control. After infected with lentiviruses, the cells were collected for verification of the knockdown efficiency by Western blot and quantitative reverse‐transcription polymerase chain reaction (qRT‐PCR) assays. The specific sequences are shown in Supporting Information Table S2. The CAG‐SETD2‐TagRFP adenoviral vector, established on the basis of the full‐length cDNA sequence of SETD2 (obtained from Professor Li Li), was then transduced into TF1‐BAR cells (10 µg vector per 1 × 10^6^ cells) using the Amaxa Nucleofector Kit (Cat# VCA‐1002; Lonza, Cologne, AG, Germany), followed by Western blot and qRT‐PCR assays to check the efficiency.

### 2.3 Cell proliferation assay

Cells were maintained in 96‐well plates (1.5 × 10^4^ cells/well) with different doses of the experimental drugs at 37°C for 72 hours, following which 10 µL of Cell Counting Kit‐8 (CCK‐8) reagent was added to each well and the plates were further incubated at 37°C in a humidified 5% CO_2_ atmosphere for 2.5‐3 hours. Finally, the absorbance at 450 nm was measured using a microplate reader.

### 2.4 Apoptosis assay

Cells were treated with the experimental drugs in 6‐well plates (1 × 10^6^ cells/well) at 37°C for 72 hours and then harvested for Annexin V (Cat#640920; Biolegend, San Diego, CA) and 7‐aminoactinomycin D (Cat#420404; Biolegend) or 4′,6‐diamidino‐2‐phenylindole (Cat#D9542; Sigma‐Aldrich, St. Louis, MO) staining. Finally, the cells were analysed on a BD FACS Canto II system (BD Biosciences, Franklin Lakes, NJ) or a C6 flow cytometer (BD Biosciences).

### 2.5 Cell cycle analysis

Cells were cultured with the experimental drugs in 6‐well plates (1 × 10^6 ^cells/well) at 37°C for 72 hours and then labelled with 10 µmol/L 5‐ethynyl‐2‐deoxyuridine (EdU) for 3 hours. Subsequently, the cells were harvested for EdU staining, using the Click‐iT EdU Flow Cytometry Assay Kit (Cat#C1024; Invitrogen, Carlsbad, CA) according to the manufacturer's instructions, and finally analysed by flow cytometry.

### 2.6 Quantitative RT‐PCR

Total RNA was isolated from the cells using the TRIzol reagent (Invitrogen) and then reverse‐transcribed to cDNA using the PrimeScript RT Reagent Kit (Takara, Tokyo, Japan) according to the manufacturer's instructions. qRT‐PCR was performed using the SYBR Premix ExTaq (Takara) and an Applied Biosystems 7500 Fast Real‐Time PCR system. The relative expression of the mRNA was analysed using the $2^{-\Delta \Delta C_{\text{t}}}$ method and normalized to the expression of β‐actin. All experiments were repeated three times. The PCR primer sequences used in this study are shown in Supporting Information Table S3.

### 2.7 Western blot assay

Cells (1 × 10^6^) were lysed in 100 µL of 2× sodium dodecyl sulphate (SDS) sample buffer containing radioimmunoprecipitation assay buffer, phenylmethylsulfonyl fluoride and protease inhibitors; sonicated for 20 seconds; and then boiled at 98°C for 8 minutes. Equal amounts of protein were then separated by SDS‐polyacrylamide gel electrophoresis and electrotransferred to polyvinylidene difluoride membranes (Millipore, Burlington, MA). After blocked with 5% not‐fat milk, the membranes were then incubated with the following primary antibodies at 4°C overnight: anti‐SETD2 (Cat#69836; Abcam, Cambridge, UK; Cat#LS‐C332416; LSBio, Seattle, WA), anti‐H3 (Cat#ab1791; Abcam), anti‐H3K36me3 (Cat#ab9050; Abcam), anti‐H3K27me3 (Cat#ab6002; Abcam), anti‐H3K79me2 (Cat#ab3594; Abcam), anti‐TBP (Cat#66166‐1‐lg; Proteintech, Chicago, IL), anti‐H3K9me3 (Cat#A2360; ABclonal, Boston, MA) and anti‐β‐actin (Cat#3700; Cell Signaling Technology, Danvers, MA).

### 2.8 Colony formation assay

Cells were maintained in methylcellulose medium (Cat#H4434; STEMCELL Technologies, Vancouver, BC, Canada) supplemented with 20 ng/mL human interleukin‐6 (Cat#200‐06‐20UG; PeproTech, Rocky Hill, NJ) and 20 ng/mL human granulocyte/colony‐stimulating factor (Cat#300‐23‐10UG; PeproTech), in 35‐mm dishes with incubation at 37°C under 5% CO_2_ and 95% humidity. After 10‐12 days, colonies were observed and counted with an inverted microscope.

### 2.9 mRNA sequencing analysis

Total RNA was extracted from the TF1‐BA and TF1‐BAR cells using TRIzol reagent (Invitrogen). Libraries were then established using the NEBNext Ultra RNA Library Prep Kit for Illumina (New England Biolabs, Ipswich, MA) according to the manufacturer's instructions. Sequencing of the libraries was established through the Illumina HiSeq platform to obtain 150‐bp paired‐end reads. After discarding adaptor sequences with poor quality, clean reads were collected and analysed using the TopHat‐Cufflinks‐Cuffmerge‐Cuffdiff pipeline with default parameters. The reads were mapped to the *Homo sapiens* GRCh38 reference genome using TopHat v2.0.13. We used DESeq2 for differential gene expression analysis, with the differentially expressed genes defining as |log_2_Foldchange| ≥ 1 and *q* value <0.05. Fold change is the ratio of the two groups after homogenization. The *q* value (padj value, the corrected *P* value) is calculated with R package (DESeq2) and adjusted by Benjamini‐Hochberg method according to the *P* value. This experiment was conducted by Annoroad Gene Tech. (Beijing) Co., Ltd. The RNA‐seq dataset was submitted into the GEO database, and the accession number is GSE124894.

### 2.10 ChIP‐quantitative PCR

To crosslink proteins to DNA, cells were incubated with 1% formaldehyde at room temperature for 10 minutes and then subjected to micrococcal nuclease treatment to digest the DNA followed by sonication to break the nuclear membrane. Finally, chromatin fragments in the range of 150‐900 bp were obtained. After analysis of the digestion and concentration, the crosslinked chromatin (10 µg) was immunoprecipitated with anti‐H3K36me3 antibodies (10 µL) (Cell Signaling Technology). All these experimental procedures were performed using the SimpleChIP Enzymatic Chromatin IP Kit (Magnetic Beads, Cat#9003; Cell Signaling Technology) according to the manufacturer's instructions. The purified DNA was analysed by qRT‐PCR using primers specific for the erythroblast transformation‐specific (ETS)‐related gene (*ERG*) and N‐myc proto‐oncogene protein (*MYCN*) oncogenes. The primer information is shown in Supporting Information Table S4.

### 2.11 Statistical analysis

Data with two groups were analysed using the unpaired Student's *t* test, and data with three or more groups were analysed by ANOVA. GraphPad Prism 7 software was used for all graphical and statistical analyses, with a *P*‐value <0.05 (*), <0.01 (**) or <0.001 (***) considered statistically significant. Experimental results are expressed as the mean ± standard deviation.

## 3 RESULTS

### 3.1 SETD2 downregulation in imatinib‐resistant cells is restored by protease inhibitor treatment

**Figure 1:**
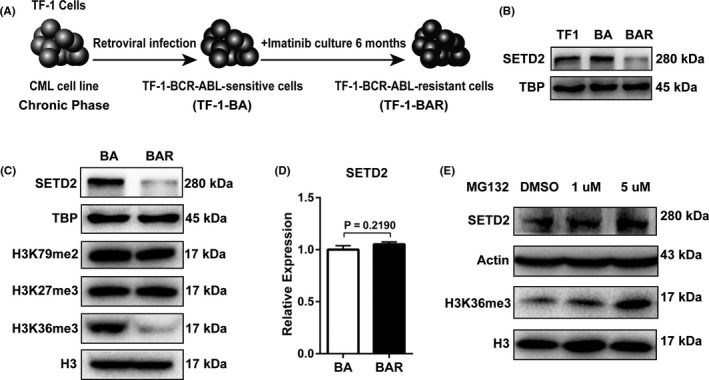
SETD2 is downregulated in imatinib (IM)‐resistant cells compared with IM‐sensitive cells. A, Establishment of the IM‐sensitive and IM‐resistant cells. TF1 cells were sensitized to IM after infection with the p210^BCR‐ABL^ retrovirus (TF1‐BA). Following 6 mo of continuous IM treatment, the cells became resistant to IM (TF1‐BAR). B, Western blot analysis of SETD2 expression level among TF1, TF1‐BA and TF1‐BAR cells. C, Western blot assays were performed to compare the protein expression levels of SETD2, H3K36me3 and associated histone‐modified sites between the TF1‐BA and TF1‐BAR cells. D, The SETD2 expression level in the TF1‐BA and TF1‐BAR cells was quantified by qRT‐PCR analysis and normalized to the β‐actin expression level. E, Western blot analysis of SETD2 and H3K36me3 expression changes due to MG132 treatment. Data are presented as the mean ± SD of three independent experiments. Data were analysed using the unpaired Student's *t* test

Imatinib‐sensitive (TF1‐BA)^44^ and imatinib‐resistant (TF1‐BAR) cell models were created by Dr Voetzel in the laboratory of Dr Veronique Maguer‐Satta, as outlined in Figure 1A. To uncover the role of SETD2 in imatinib resistance development in CML, we first analysed the SETD2 expression level in the TF1, TF1‐BA and TF1‐BAR cells by Western blot and qRT‐PCR assays. The results showed that P210^BCR‐ABL^ did not cause a change of SETD2 expression level (Figure 1B), and the SETD2 protein expression level was significantly lower in the TF1‐BAR cells than in the TF1‐BA cells and, likewise, the levels of H3K36me3. However, the other histone modification sites were not influenced (Figure 1C) and the mRNA level of *SETD2* was unchanged (Figure 1D). Additionally, the downregulation of both SETD2 and H3K36me3 in the TF1‐BAR cells could be successfully restored by protease inhibitor (MG132) treatment (Figure 1E). These data indicated that the SETD2 deficiency was associated with imatinib resistance.

### 3.2 SETD2 facilitates imatinib sensitivity in CML cell lines

**Figure 2:**
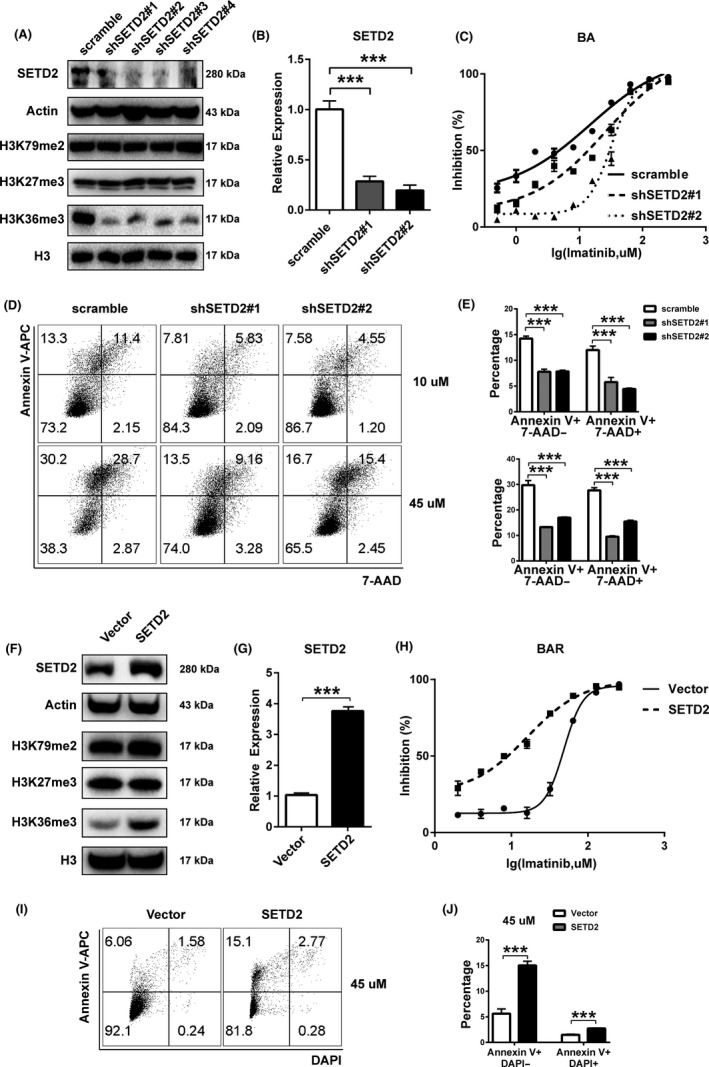
SETD2 facilitates imatinib sensitivity in CML cell lines. A, B, Protein expression levels of SETD2, H3K36me3 and related histone‐modified sites (A) and mRNA expression level of SETD2 (B) in TF1‐BA cells transfected with different *SETD2*‐targeted shRNA. C, IC_50_ curves of imatinib (IM)‐treated SETD2‐KD‐BA cells. D, E, Flow cytometric assay of the apoptotic percentage of IM‐treated SETD2‐KD‐BA cells. F, G, Protein expression levels of SETD2, H3K36me3 and relevant histone‐modified sites (F) and mRNA expression level of SETD2 (G) in SETD2‐OE‐BAR cells. H, IC_50_ curves of IM‐treated SETD2‐OE‐BAR cells. I, J, Flow cytometric assay of the apoptotic percentage of IM‐treated SETD2‐OE‐BAR cells. Data are presented as the mean ± SD of at least three independent experiments. ***P < 0.001, by ANOVA

To further investigate the role of SETD2 in the process of imatinib resistance acquisition by CML cells, we next used *SETD2*‐targeted shRNA to knock down the gene in TF1‐BA cells. The knockdown efficiency and the consistent downregulated expression of H3K36me3 were checked by Western blot and qRT‐PCR assays (Figure 2A,B). We also overexpressed SETD2 in TF1‐BAR cells (SETD2‐OE‐BAR) through electroporation with an *SETD2*‐overexpressing plasmid, where the transfection efficiency and corresponding increased expression of H3K36me3 were also determined by Western blot and qRT‐PCR assays (Figure 2F,G). According to the cell viability assays, the SETD2‐KD‐BA cells were more resistant to imatinib treatment than the TF1‐BA cells were (Figure 2C), whereas the SETD2‐OE‐BAR cells were more sensitive to imatinib than the TF1‐BAR cells were (Figure 2H). SETD2 downregulation in the TF1‐BA cells resulted in reduced apoptosis (Figure 2D,E), whereas SETD2 overexpression in the TF1‐BAR cells caused an increase in apoptosis (Figure 2I,J), in cells treated with imatinib. We obtained consistent results using the KCL‐22 cell lines, where SETD2 knockdown induced imatinib resistance and decreased the apoptotic percentage (Supporting Information Figure S1C‐G) in the KCL‐22‐S cells with endogenous BCR‐ABL expression. Collectively, we could conclude that SETD2 facilitates imatinib sensitivity in the CML cell lines through the increase in apoptotic events, having no influence on the cell cycle (Supporting Information Figure S2).

### 3.3 SETD2 deficiency upregulates the CD34^+^CD38^−^ stem cell percentage and quantity

**Figure 3:**
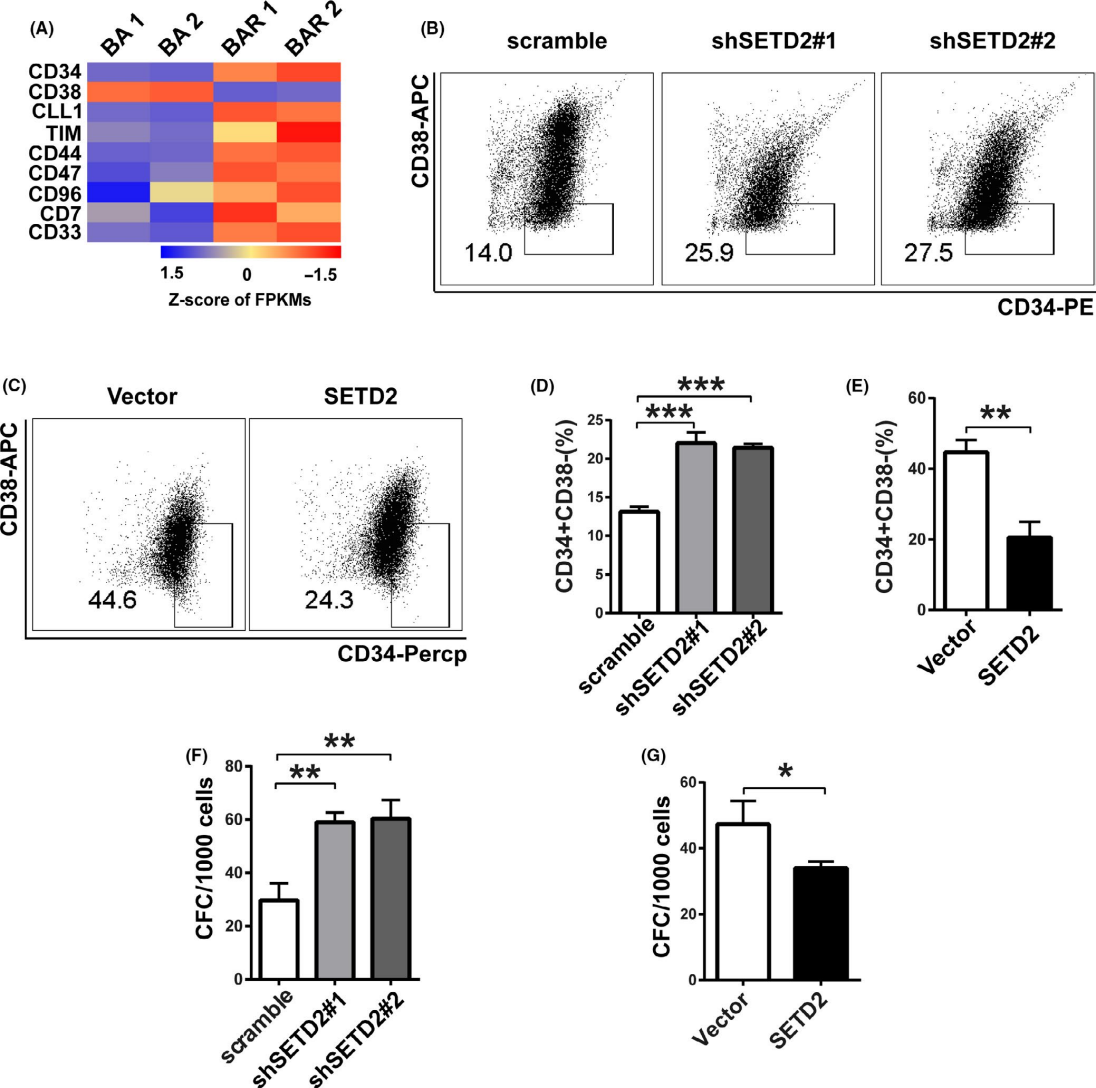
SETD2 deficiency upregulates the CD34^+^CD38^−^ stem cell percentage and quantity. A, Heatmap of differentially expressed stem cell markers in TF1‐BA and TF1‐BAR cells, as analysed by RNA sequencing. FPKMs, fragments per kilobase of transcript per million mapped reads. B, D, Flow cytometric assay of the CD34^+^CD38^−^ stem cell percentage of SETD2‐KD‐BA cells. C, E, Flow cytometric assay of the CD34^+^CD38^−^ stem cell percentage of SETD2‐OE‐BAR cells. F, Colony formation assay of the quantity of stem cells in SETD2‐KD‐BA cells. G, Colony formation assay of the quantity of stem cells in SETD2‐OE‐BAR cells. Data are presented as the mean ± SD of three independent experiments. *P < 0.05, **P < 0.01, ***P < 0.001, by ANOVA

BCR‐ABL tyrosine kinase point mutations and LSC outgrowth are the two main events resulting in imatinib resistance and CML propagation.^14^, ^20^, ^21^ For the BCR‐ABL tyrosine kinase mutation analysis, we performed nest PCR to amplify the ABL kinase domain of BCR‐ABL gene using genomic DNA extracted from TF1‐BAR and KCL‐22‐R cells. The PCR products were purified for sequencing. The DNA sequence of the ABL kinase domain was aligned to the ABL kinase ref. sequence (NM_005157.5). We did not find any kinase mutation in TF1‐BAR and KCL‐22‐R imatinib‐resistant cells, indicating that stem cell‐associated changes might have occurred during the acquisition of imatinib resistance by the CML cell lines. RNA sequencing was performed to quantify some stem cell markers in the TF1‐BA and TF1‐BAR cells, whereupon the markers CD34, CLL1, TIM, CD44, CD47, CD96, CD7 and CD33 were found to be significantly upregulated, whereas CD38 was downregulated in the TF1‐BAR cells, which correlated positively to leukaemic stemness^45^, ^46^ (Figure 3A). Flow cytometric analysis showed a high percentage of CD34^+^CD38^−^ markers in the SETD2‐KD‐BA cells (Figure 3B,D) and a low percentage in the SETD2‐OE‐BAR cells (Figure 3C,E). These results were consistent with the colony formation assay (CFC) results for both cell lines, where the SETD2‐KD‐BA cells formed more colonies induced by SETD2 knockdown and SETD2‐OE‐BAR cells formed less colonies induced by SETD2 overexpression (Figure 3F,G). Taken together, the results indicated that SETD2 negatively regulates the CD34^+^CD38^−^ stem cell percentage and quantity in CML.

### 3.4 SETD2 deficiency upregulates a subset of genes involved in stemness regulation

**Figure 4:**
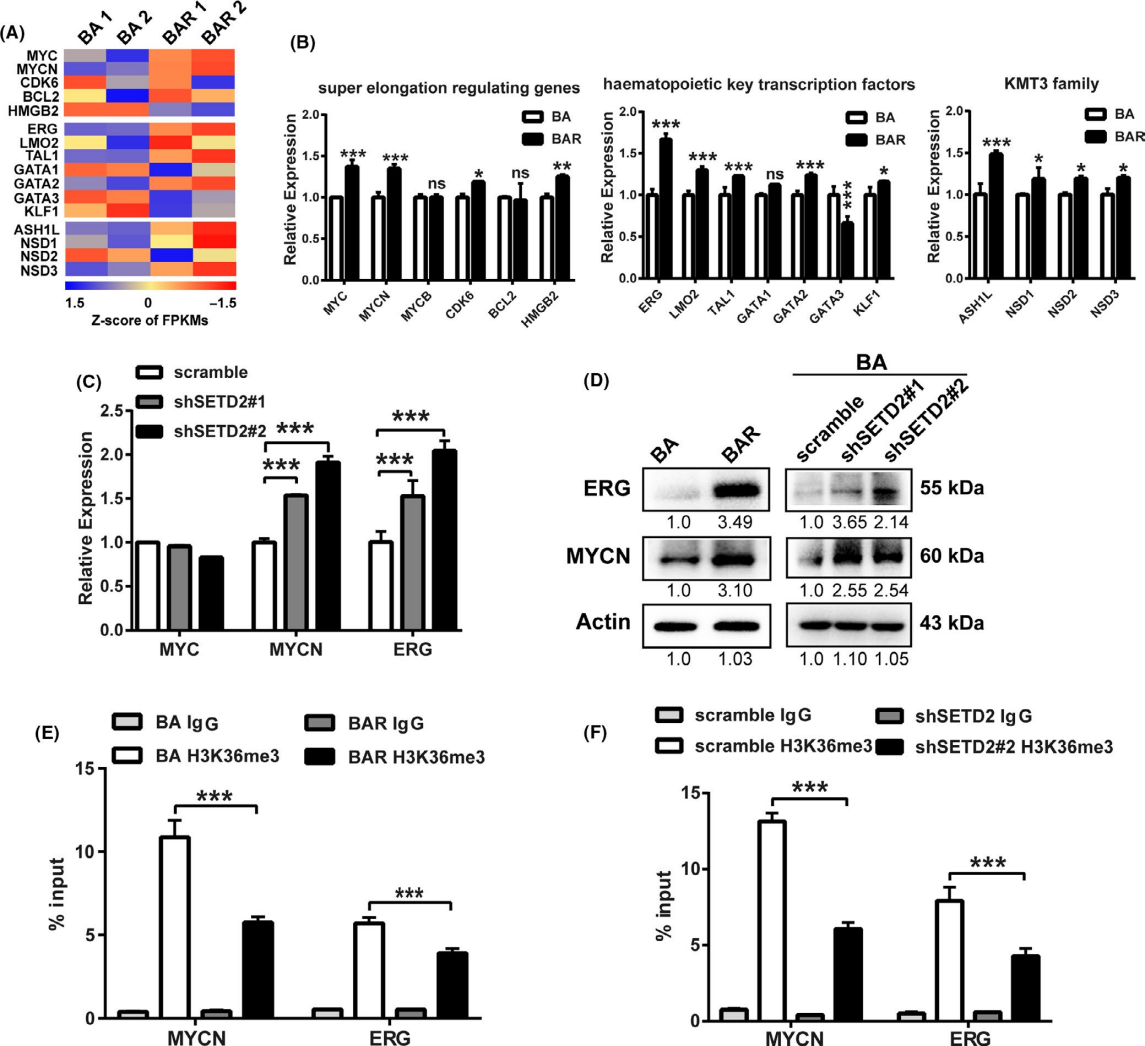
SETD2 downregulation promotes higher ERG and MYCN expression. A, B, Heatmaps of the mRNA expression of super elongation‐regulating genes, key hematopoietic transcription factor genes and KMT family genes in TF1‐BA and TF1‐BAR cells, FPKMs, fragments per kilobase of transcript per million mapped reads (A) and qRT‐PCR verification of the results (B). C, qRT‐PCR analysis of the *MYC*, *MYCN* and *ERG* mRNA expression levels in SETD2‐KD‐BA cells. D, Western blot assay of the ERG and MYCN protein expression levels in TF1‐BA and TF1‐BAR cells (left) and SETD2‐KD‐BA cells (right). (E, F) ChIP‐quantitative PCR assay of the H3K36me3 level on the *ERG*, and *MYCN* gene bodies in TF1‐BA and TF1‐BAR cells (E) and SETD2‐KD‐BA cells (F). Data are presented as the mean ± SD. *P < 0.05, **P < 0.01, ***P < 0.001, by ANOVA

To further study how SETD2 deficiency confers the upregulation of stem cells in imatinib‐resistant CML cell lines, we performed RNA sequencing on the TF1‐BA and TF1‐BAR cells. Genes coding for proteins involved in super elongation regulation, hematopoietic key transcription factors and the KMT3 family members, which were reported to participate in the dysfunction of hematopoietic stem cells and their malignant transformation caused by SETD2 depletion,^43^ were shown to be upregulated at the mRNA level in the TF1‐BAR cells compared with that in the TF1‐BA cells (Figure 4A). These results were verified by qRT‐PCR (Figure 4B). Among the genes that were upregulated in the TF1‐BAR cells, *ERG* and *MYCN* were significantly upregulated in the SETD2‐KD‐BA cells, as shown by qRT‐PCR (Figure 4C). Western blot assays were also performed to verify the high protein expression of ERG and MYCN in the SETD2‐deficient cells (Figure 4D). The upregulated expression of ERG and MYCN, which are important transcription factors that are reported to be positively related to poor clinical outcomes in patients with acute myeloid leukaemia and acute lymphocytic leukaemia,^40^, ^47^, ^48^, ^49^, ^50^, ^51^ seemed to induce the LSC accumulation mediated by SETD2 deficiency. ChIP‐quantitative PCR was performed to further explore whether the changes in ERG and MYCN expression were due to SETD2‐associated histone modification alterations. As a result, lower H3K36me3 levels were detected at the ERG and MYCN gene bodies in the imatinib‐resistant cells (Figure 4E,F). This indicated that the lower levels of H3K36me3 modification in the SETD2‐deficient cells had contributed to ERG and MYCN activation, which caused LSC enhancement.

### 3.5 *ERG* and/or *MYCN* knockdown successfully reverses the effects of SETD2 knockdown

**Figure 5:**
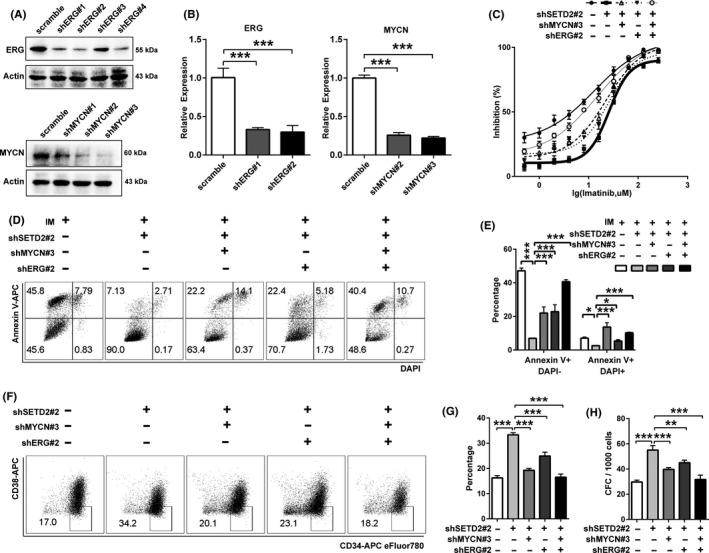
Downregulation of both ERG and MYCN restores imatinib (IM) sensitivity in *SETD2*‐knockdown TF1‐BA cells. A, B, Western blot (A) and qRT‐PCR (B) assays of the efficiency of ERG and MYCN knockdown in SETD2‐KD‐BA cells. C, IC_50_ curves of the sensitivity of SETD2‐KD‐BA cells to IM after ERG and MYCN knockdown. D, E, Flow cytometric assay of the apoptosis of IM‐treated SETD2‐KD‐BA cells after ERG and MYCN knockdown. F, G, Flow cytometric assay of the effect of ERG and MYCN knockdown on the CD34^+^CD38^−^ stem cell percentage in SETD2‐KD‐BA cells. H, Colony formation assay of the effect of ERG and MYCN knockdown on the CD34^+^CD38^−^ stem cell quantity in SETD2‐KD‐BA cells. Data are presented as the mean ± SD of three independent experiments. *P < 0.05, **P < 0.01, ***P < 0.00, by ANOVA

To determine the functional relevance of ERG and MYCN with SETD2 knockdown, we used shRNAs to knock down *ERG* and *MYCN* individually or together in SETD2‐KD‐BA cells, and detected the knockdown efficiency by Western blot (Figure 5A) and qRT‐PCR assays (Figure 5B). *ERG* and/or *MYCN* knockdown successfully rescued the imatinib resistance effect caused by SETD2 knockdown in the SETD2‐KD‐BA cells, where a lower cell survival rate was shown in the IC_50_ experiment (Figure 5C). The increased apoptotic percentage obtained by flow cytometric analysis (Figure 5D,E) also reflected that imatinib sensitivity had been restored in the SETD2‐KD‐BA cells after *ERG* and/or *MYCN* knockdown. Additionally, *ERG* and/or *MYCN* knockdown abrogated increased percentage of CD34^+^CD38^−^ stem cells determined by flow cytometry (Figure 5F,G) and higher number of colonies obtained in the colony formation assay (Figure 5H) induced by SETD2 knockdown in TF1‐BA cells. These data all revealed the key roles of ERG and MYCN in the propagation of CML cells with acquired imatinib resistance as SETD2 targets modulated by H3K36me3 alterations.

### 3.6 Small‐molecule inhibitor targeting H3K36me3 loss partially reverses the imatinib resistance

**Figure 6:**
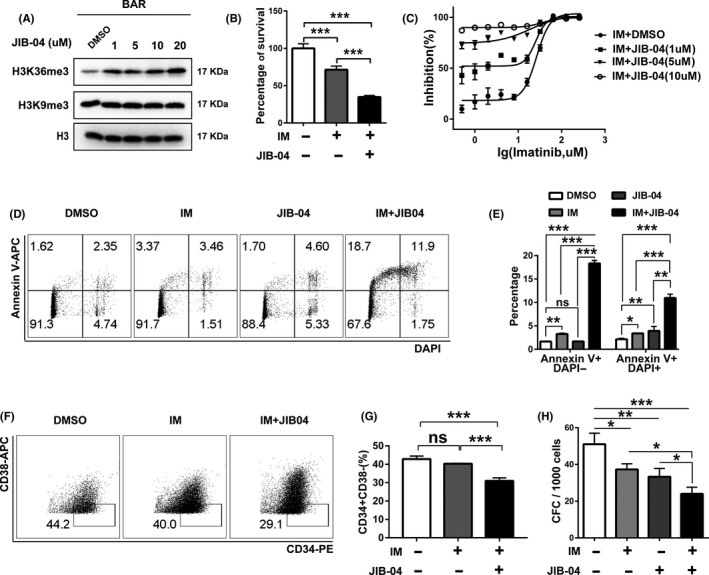
JIB‐04, a H3K36me3 demethylase, restores the H3K36me3 level and promotes imatinib (IM) sensitivity in TF1‐BAR cells. A, Western blot assay of H3K36me3 and H3K9me3 expression changes in TF1‐BAR cells after treatment with different doses of JIB‐04 for 72 h. B, C, CCK‐8 assay and IC_50_ curves of the effects of JIB‐04 alone or in combination with IM on TF1‐BAR cells. D, E, Flow cytometric assay of the apoptosis of TF1‐BAR cells after JIB‐04 (1 μmol/L) and/or IM treatment for 72 h. F, G, Flow cytometric assay of the percentage of CD34^+^CD38^−^ stem cells of TF1‐BAR cells after JIB‐04 (1 μmol/L) and/or IM treatment for 72 h. H, Colony formation assay of the effects of JIB‐04 (1 μmol/L) and/or IM treatment on the quantity of stem cells in TF1‐BAR cells. Bars in the graphs represent the mean ± SD. *P < 0.05, **P < 0.01, ***P < 0.001, by ANOVA

Given that the loss of SETD2 leads to the acquisition of imatinib resistance in TF1‐BAR cells, we hypothesized that improving the expression level of H3K36me3 might restore the imatinib sensitivity of these cells, in view of the facts that SETD2 is the major tri‐methyltransferase of H3K36 and the corresponding downregulation of H3K36me3 occurs after SETD2 deficiency. Thus, inhibiting the demethylation of H3K36me3 might be a good compensation for the lowered trimethylation state. The small molecule JIB‐04 would be a promising therapeutic drug for such inhibition of various histone demethylases, including KDM4A,^52^ a demethylase of H3K9 and H3K36. Through analysing the RNA‐seq data, we found no significant alterations of KDM4A, KDM1A and KDM2A in imatinib‐resistant vs imatinib‐sensitive CML cells, indicating that the downregulation of H3K36me3 is mainly caused by SETD2 deficiency rather than histone demethylases. Therefore, we incubated TF1‐BAR cells with different doses of JIB‐04 for 72 hours and then measured the change of H3K36me3 expression by Western blot assay (Figure 6A). The results showed that JIB‐04 had significantly restored the expression level of H3K36me3 without changing the status of H3K9me3. This may be caused by differential sensitivity of histone demethylation inhibition on H3K36me3 and H3K9me3 upon JIB‐04 treatment in imatinib‐resistant CML cells. When combined with imatinib, JIB‐04 exerted a synergetic effect in promoting imatinib sensitivity in the TF1‐BAR cells, as demonstrated by the minimal cell survival rate, the increased inhibition rate (Figure 6B,C) and the facilitated apoptosis (Figure 6D,E). Combination treatment of JIB‐04 and imatinib resulted in a decrease of CD34^+^CD38^−^ stem cell percentage (Figure 6F,G) and CFC number (Figure 6H). The enhanced effects of imatinib and JIB‐04 combined on the imatinib‐resistant cells were also evident in KCL‐22‐R cells (Supporting Information Figure S3). These studies illustrated that therapies that target H3K36me3 deficiency might be of great value in the treatment of imatinib resistance due to SETD2 loss.

## 4 DISCUSSION

In our study, we discovered that SETD2 deficiency significantly promoted both imatinib resistance and LSC marker upregulation in CML cells. JIB‐04 could efficiently improve the sensitivity of CML cells to imatinib by inhibiting the biological process of histone demethylation, indicating it as a promising therapeutic agent for imatinib‐resistant CML.

SETD2, an important histone modifier responsible for producing H3K36me3,^32^ participates in many of the biological processes for maintaining mitosis and genomic stability, namely identification of the splicing machinery and selection of splicing sites^53^; elongation of RNA polymerase and prevention of spurious transcriptional initiation^54^; recognition of DNA repair protein and activation of mismatch repair and homologous recombination^55^, ^56^; and remoulding of chromatin and the cytoskeleton through tubulin methylation.^57^, ^58^ Although the genetic disruptions of *SETD2* have been validated in various haematological malignancies, including acute myeloid leukaemia, acute lymphocytic leukaemia and chronic lymphocytic leukaemia,^38^, ^39^ whether these genetic changes are involved in CML had remained unsolved. Our finding that the SETD2 deficiency occurs at the post‐translational level, with unchanged mRNA expression, during imatinib resistance in CML provides evidence that in addition to genetic changes, the post‐translational mechanisms also play a vital role in promoting the progression of leukaemia triggered by SETD2 dysregulation. However, whether SETD2 is mutated in patients with CML, especially those who harboured IM resistance, needs to be further studied.

The repression, but not total loss, of SETD2 facilitated the imatinib resistance of CML, which is consistent with the finding that the heterozygous depletion of SETD2 accelerated MLL‐AF9 leukaemia propagation, whereas its homozygous depletion greatly dampened the latency.^41^ Another interesting fact is that the homozygous depletion of SETD2 is detrimental to hematopoietic stem cells,^42^, ^43^ and thus, we speculate that the moderate expression of SETD2 is required for maintenance of the stemness in LSCs that is mediated by the upregulation of oncogenic transcription factors such as MYCN and ERG, both of which accelerate the development of acute leukaemia and lead to poor patient outcomes.^47^, ^48^, ^49^, ^50^, ^51^

MYCN and MYC both play essential roles in tumorigenesis,^49^, ^50^, ^59^, ^60^ and it is intriguing that the upregulation of MYCN instead of MYC induced by H3K36me3 reduction conferring imatinib resistance. Consistently, MYCN upregulation displays a strong positive correlation with the poor outcomes of acute leukaemia patients.^49^, ^50^, ^51^ The differential impacts of H3K36me3 downregulation on MYCN and MYC await further investigation.

The proliferation and accumulation of LSCs remain the leading cause of imatinib resistance in CML, despite the ongoing efforts that have been made to treat the disease. Thus, better clarification of the mechanisms underlying the pathogenesis triggered by LSCs and the identification of novel small molecules that can specifically eradicate LSCs are critical for the clinical treatment of patients with CML, especially those with imatinib resistance. Continuing advances in epigenetics have indicated that the histone modifiers associated with imatinib‐resistant CML are potential therapeutic targets, with several histone deacetylase inhibitors cited as attractive drug candidates already under clinical development for therapeutic interventions.^20^, ^31^ In the present study, we found that the small molecule JIB‐04, an inhibitor of several demethylases, could efficiently eliminate LSCs through the apoptosis triggered by correction of the imbalanced epigenetics to restore the level of H3K36me3. JIB‐04 therefore has potential for broader clinical applications, especially in imatinib resistance treatment.

Taken together, the results of our study have built an oncogenic role for SETD2 downregulation in CML with imatinib resistance. Nonetheless, because we only measured these biological effects using in vitro experiments, further in vivo verification of these effects is needed as well as validation in clinical samples. Importantly, however, this is the first report to suggest that targeting H3K36me3 deficiency through the use of JIB‐04 could be a novel strategy to improve clinical treatments for imatinib resistance. Our study paves the way for utilizing SETD2 to identify potential diagnostic strategies or drug targets to eventually cure CML, especially in patients with imatinib resistance.

## CONFLICT OF INTEREST

The authors declare no conflicts of interest exist.

## AUTHOR CONTRIBUTIONS

HHZ designed the research plan and interpreted the data; YS performed experiments and analysed data; W‐QG and VM‐S assisted in paper writing and editing; TV established the cell models; ZJ, WX and HZ helped to conduct the plasmid construction; ZJ and JW assisted with RNA sequencing analysis; HHZ and YS wrote the manuscript; CC, XW, YH and KL assisted in experiments.
