# Supplementary figures and images for: Downregulation of the histone methyltransferase SETD2 promotes imatinib resistance in chronic myeloid leukaemia cells
Source: Cell Prolif. 2019 May 3;52(4):e12611. doi: 10.1111/cpr.12611 (PMC6668982; doi:10.1111/cpr.12611)

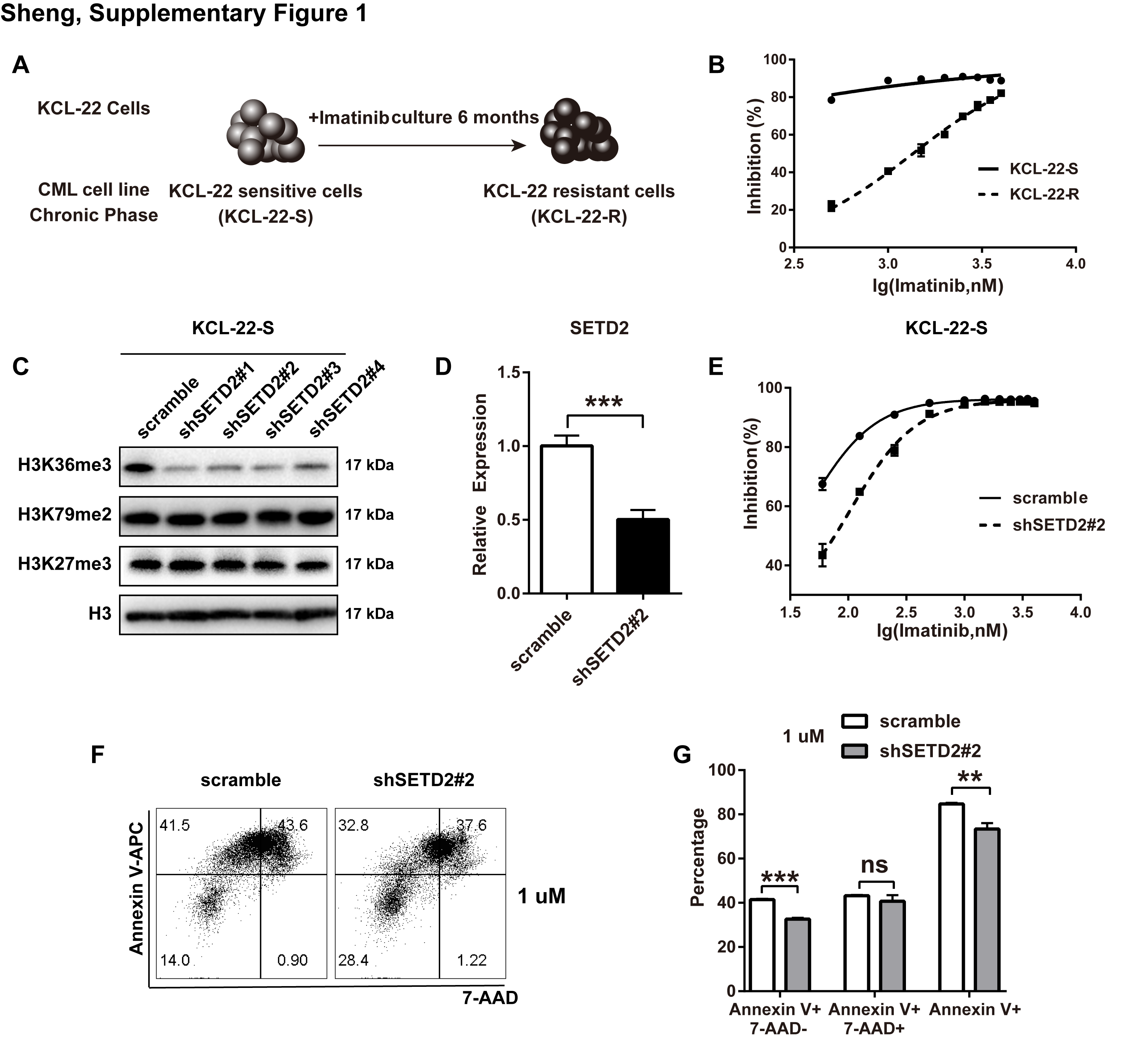

Supplement: Supplementary file 1 [file CPR-52-e12611-s001.tif]

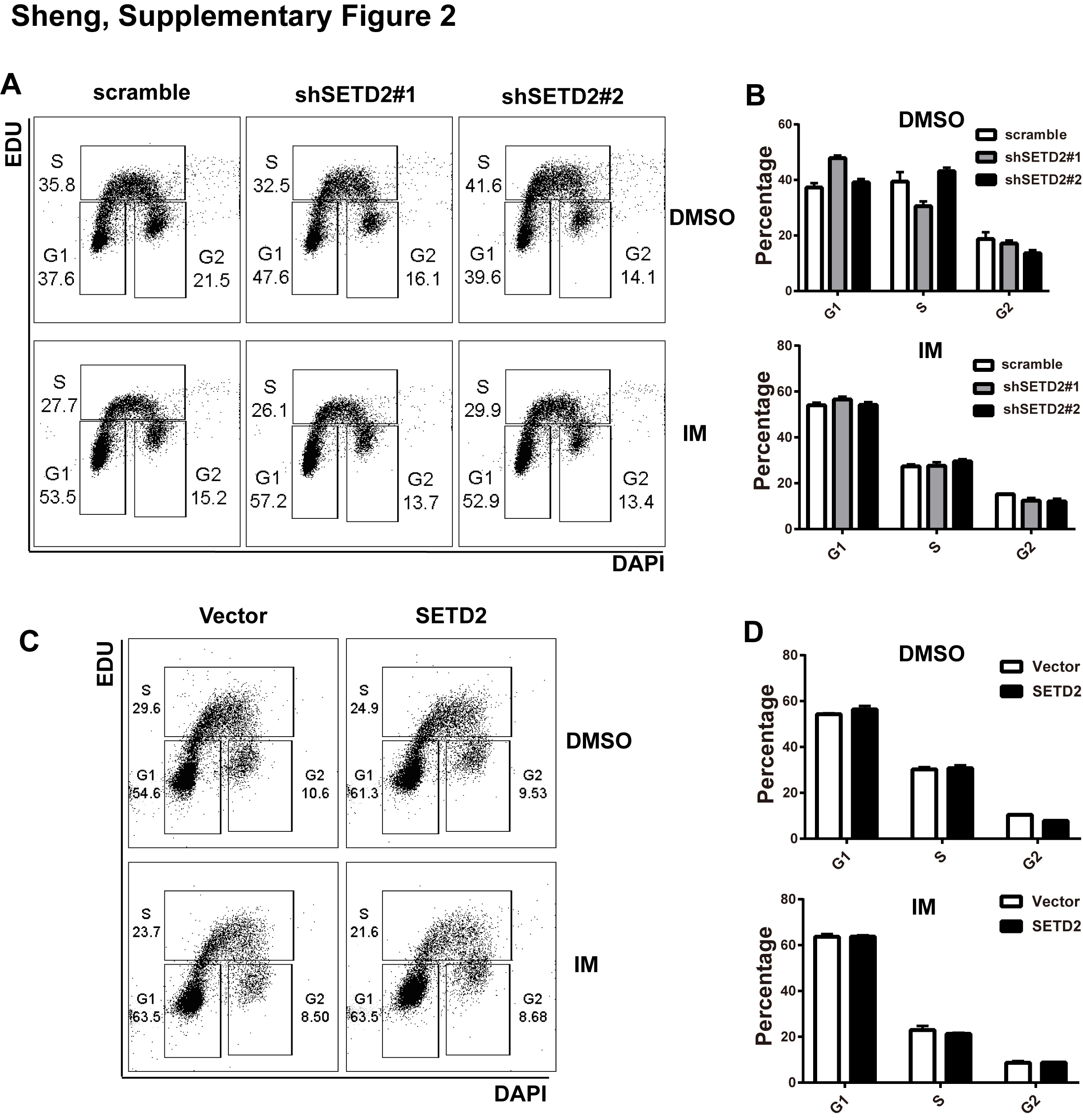

Supplement: Supplementary file 2 [file CPR-52-e12611-s002.tif]

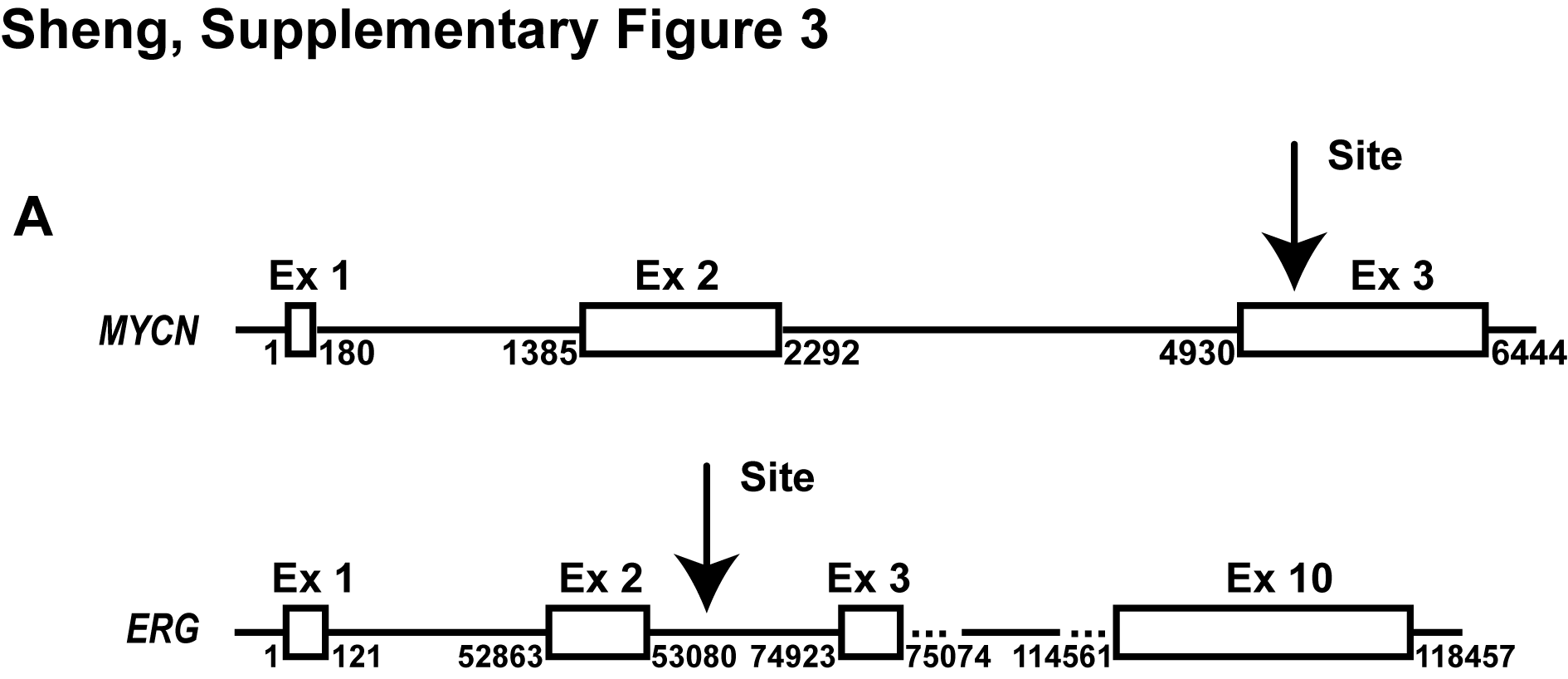

Supplement: Supplementary file 3 [file CPR-52-e12611-s003.tif]

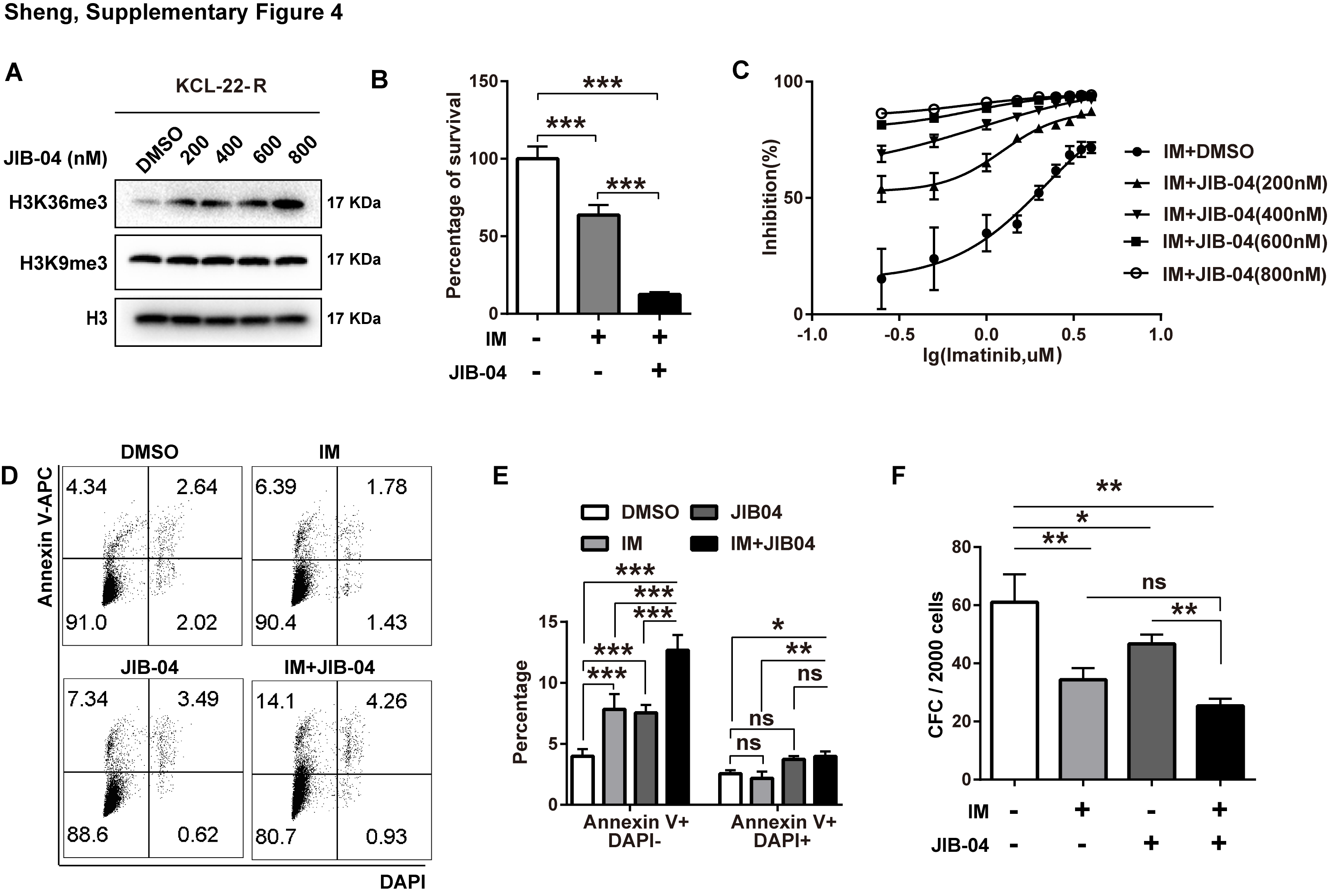

Supplement: Supplementary file 4 [file CPR-52-e12611-s004.tif]
