# Supplementary material for: Downregulation of the histone methyltransferase SETD2 promotes imatinib resistance in chronic myeloid leukaemia cells
Source: Cell Prolif. 2019 May 3;52(4):e12611. doi: 10.1111/cpr.12611 (PMC6668982; doi:10.1111/cpr.12611)
Supplement: Supplementary file 5 [file CPR-52-e12611-s005.docx]

**Supplementary tables**

| **Supplementary Table 1: reagents related in this study.** | | |
| --- | --- | --- |
| **Reagents** | **Source** | **Cataloge Number** |
| Imatinib Mesylate | MedChemExpress | HY-50946 |
| JIB-04 | MedChemExpress | HY-13953 |

| **Supplementary Table 2: shRNA sequences in this study.** | | |  |
| --- | --- | --- | --- |
| **shRNA** | | **sense (5-3)** | **antisense (5-3)** |
| sh-SETD2#1 | AAGCAGGACACTATATCTAAT | ATTAGATATAGTGTCCTGCTT |  |
| sh-SETD2#2 | ATGGTGTAACTTATGCATTAA | TTAATGCATAAGTTACACCAT |  |
| sh-SETD2#3 | ATCCCGGCTAATGGTTAGAAT | ATTCTAACCATTAGCCGGGAT |  |
| sh-SETD2#4 | TGCCCTATGACTCTCTTGGTT | AACCAAGAGAGTCATAGGGCA |  |
|  |  |  |  |
| sh-MYCN#1 | CAGTATTAGACTGGAAGTT | AACTTCCAGTCTAATACTGGC |  |
| sh-MYCN#2 | GCAGCAGTTGCTAAAGAAA | TTTCTTTAGCAACTGCTGCTG |  |
| sh-MYCN#3 | GTCGCAGAAACCACAACAT | ATGTTGTGGTTTCTGCGACGC |  |
|  |  |  |  |
| sh-ERG#1 | ATCTGGGCACTTACTACTAAA | TTTAGTAGTAAGTGCCCAGAT |  |
| sh-ERG#2 | GAGACTCCTCTTCCACATTTG | CAAATGTGGAAGAGGAGTCTC |  |
| sh-ERG#3 | CCGTTACTACTATGACAAGAA | TTCTTGTCATAGTAGTAACGG |  |

| **Supplementary Table 3: Primers for qRT-PCR in this study.** | |
| --- | --- |
| **Primer** | **Sequences** |
| SETD2-F | TGCTTCTAGTCGATTTTTGCCC |
| SETD2-R | AGGGTTTGGAGTATCACTTTGC |
| MYC-F | GGCTCCTGGCAAAAGGTCA |
| MYC-R | CTGCGTAGTTGTGCTGATGT |
| MYCN-F | TGATCCTCAAACGATGCCTTC |
| MYCN-R | GGACGCCTCGCTCTTTATCT |
| MYCB-F | ATCTCCCGAATCGAACAGATGT |
| MYCB-R | TGCTTGGCAATAACAGACCAAC |
| CDK6-F | CCAGATGGCTCTAACCTCAGT |
| CDK6-R | AACTTCCACGAAAAAGAGGCTT |
| BCL2-F | GGTGGGGTCATGTGTGTGG |
| BCL2-R | CGGTTCAGGTACTCAGTCATCC |
| HMGB2-F | GCTCGCTATGACAGGGAGATG |
| HMGB2-R | GCGATGTTCAGAGCAAAACAGG |
| ERG-F | CGTGCCAGCAGATCCTACG |
| ERG-R | GGTGAGCCTCTGGAAGTCG |
| LMO2-F | GGCCATCGAAAGGAAGAGCC |
| LMO2-R | GGCCCAGTTTGTAGTAGAGGC |
| TAL1-F | CCAAAGTTGTGCGGCGTATC |
| TAL1-R | CAGGCGGAGGATCTCATTCTT |
| GATA1-F | TTGTCAGTAAACGGGCAGGTA |
| GATA1-R | CTTGCGGTTTCGAGTCTGAAT |
| GATA2-F | GCAACCCCTACTATGCCAACC |
| GATA2-R | CAGTGGCGTCTTGGAGAAG |
| GATA3-F | GCCCCTCATTAAGCCCAAG |
| GATA3-R | TTGTGGTGGTCTGACAGTTCG |
| KLF1-F | GGTTGCGGCAAGAGCTACA |
| KLF1-R | GTCAGAGCGCGAAAAAGCAC |
| ASH1L-F | ACACTGTCCTTCAAAACGAGAC |
| ASH1L-R | GAAGAGTAGATGGCGTTGCATTA |
| NSD1-F | GAGCTACCTGTCCTTAGGAGAA |
| NSD1-R | GACTCAGGATCATTTGTGCAGT |
| NSD2-F | GCCAAACTGCGTTTTGAGTCC |
| NSD2-R | TGTTCCTTCTCGCCTTGTTTTC |
| NSD3-F | TTCCAAACACAAGACCACATGA |
| NSD3-R | ACTCAAACAATTCCCTGCCATTC |

| **Supplementary Table 4: Primers for ChIP-quantitative-PCR.** | |
| --- | --- |
| **Primer** | **Sequences** |
| ERG-F | AGCTGGTTTTAGTTTGGCCACACA |
| ERG-R | GATCAAACAGCAGCATTCTTGC |
| MYCN-F | AAGAACGCAGCCCTGGGTC |
| MYCN-R | GCTCAAGCTCTTAGCCT |
